# Spatiotemporal Pattern of Fine Particulate Matter and Impact of Urban Socioeconomic Factors in China

**DOI:** 10.3390/ijerph16071099

**Published:** 2019-03-27

**Authors:** Tuo Shi, Miao Liu, Yuanman Hu, Chunlin Li, Chuyi Zhang, Baihui Ren

**Affiliations:** 1 CAS Key Laboratory of Forest Ecology and Management, Institute of Applied Ecology, Chinese Academy of Sciences, No. 72, Wenhua Road, Shenyang 110016, China; tuoshi0411@163.com (T.S.); huym@iae.ac.cn (Y.H.); lichunlin@iae.ac.cn (C.L.); trulyzhang@foxmail.com (C.Z.); renbaihui1004@126.com (B.R.); 2 College of Resources and Environment, University of Chinese Academy of Sciences, No. 19, Yuquan Road, Beijing 100049, China; 3 Department of Geography & Planning, University of Toronto, 3359 Mississauga Road, Mississauga, ON L5L 1C6, Canada; 4 Department of Horticulture, Shenyang Agricultural University, No.120, Dongling Road, Shenyang 110866, China

**Keywords:** fine particulate matter, socioeconomic factor, land use, distribution pattern

## Abstract

Frequent hazy weather has been one of the most obvious air problems accompanying China’s rapid urbanization. As one of the main components of haze pollution, fine particulate matter (PM_2.5_), which severely affects environmental quality and people’s health, has attracted wide attention. This study investigated the PM_2.5_ distribution, changing trends and impact of urban factors based on remote-sensing PM_2.5_ concentration data from 2000 to 2015, combining land-use data and socioeconomic data, and using the least-squares method and structural equation model (SEM). The results showed that the high concentration of PM_2.5_ in China was mainly concentrated in the eastern part of China and Sichuan Province. The trends of the PM_2.5_ concentration in eastern part and Northeast China, Sichuan, and Guangxi Provinces were positive. Meanwhile, the ratios of increasing trends were strongest in built-up land and agricultural land, and the decreasing trends were strongest in forest and grassland, but the overall trends were still growing. The SEM results indicated that economic factors contributed most to PM_2.5_ pollution, followed by demographic factors and spatial factors. Among all observed variables, the secondary industrial GDP had the highest impact on PM_2.5_ pollution. Based on the above results, PM_2.5_ pollution remains an important environmental issue in China at present and even in the future. It is necessary for decision-makers to make actions and policies from macroscopic and microscopic, long-term and short-term aspects to reduce pollution.

## 1. Introduction

China is fast becoming an example of an inevitable trend towards increasing urbanization, the level of which increased from 26.41% in 1990 to 49.95% in 2010 and is projected to exceed 60% by 2020 [1]. One feature of urbanization is the rapid development of industrialization, and industrial activities emit many kinds of pollutants. Another feature of urbanization is the flow of the population from the countries to cities. Anthropogenic activities, such as the use of motor vehicles and coal fires, also contribute to air pollution.

Air pollution is a major environmental health problem affecting everyone in developed and developing countries alike. It was estimated in 2012 to cause three million premature deaths per year in urban and rural areas worldwide; the mortality is because of exposure to small particulate matter with a diameter of 10 microns or less [2], which causes cardiovascular and respiratory disease and cancers. The World Health Organization (WHO) reported that over 80% of urban residents are exposed to air quality levels of PM_2.5_ that exceed WHO limits (10 µg/m^3^) on a global scale. The China Meteorological Administration reported that, in 2013, 25 provinces and more than 100 large and medium cities were affected by haze for 29.9 days on average. Meanwhile, with the promotion of monitoring techniques and the popularization of monitoring stations, primary air pollutants have changed from traditional pollutants (e.g., NO_2_, SO_2_) to new pollutants (e.g., PM_2.5_/PM_10_) [3]. In addition, fine particulate pollution seriously affects people’s daily travel and health [4]. The air quality and people’s wellbeing are therefore closely related in urban areas.

City is a land type that has high population and changes rapidly. Due to the severe haze weather that lots of cities worldwide and in China were troubled, it is of great significance to explore the relationship between urban factors and atmospheric conditions for comprehensive management of the regional environment. Anthropogenic indicators related to social and economic processes are often analyzed, including urban population density, number of private vehicles, energy consumption, etc. [5]. Han selected built-up land, urban population and secondary industry fraction to examine the effects of urbanization on PM_2.5_ concentration; significant positive correlations were found between urban PM_2.5_ and urban population and urban second industry fraction, suggesting that urbanization had a considerable impact on PM_2.5_ concentrations [6]. Fang employed Ordinary Least Squares, Spatial Lag Model, and Geographically Weighted Regression to quantitatively estimate the comprehensive impact and spatial variations of China’s urbanization process on air quality, and revealed that urbanization has played an important negative role in determining air quality in Chinese cities. The population, urbanization rate, automobile density, and the proportion of secondary industry were all found to have a significant influence over air quality [7].

However, interaction of different urban factors and comprehensive performance of urban factors was less considered. The structural equation model (SEM) is a useful statistical approach to ascertain the driving factors of pollution, and the it was increasingly used in ecology as multivariate analysis that can represent theoretical variables and address complex sets of hypotheses [8]. For example, Fu (2018) used SEM to quantify the contribution of city size, industrial activities, residents’ activities and urban greening on atmospheric visibility in Xiamen City, China [9]. Zhao using the SEM to quantified the contributing effects of various forces driving SO_2_ and NO_X_ pollution in 2015 in prefecture-level cities of China [10]. The SEM combines a set of linear equations in a path model that not only represents the hypothetical causal relationships among selected driving factors but also calculates the direct or indirect theoretical causal relationships between driving forces and air pollution [8], which is a suitable methodology for the study.

Based on the above, we cannot help but think about a few questions, such as, how is the spatial distribution of PM_2.5_ pollution in China? Is the severe pollution concentrated in large and medium cities? What are the pollution conditions of different land use types? Are cities the main source of PM_2.5_ pollutants? Therefore, the objectives of this paper were (1) to reveal the mean spatial pattern and changing trends of the PM_2.5_ concentration from 2000 to 2015 in China, (2) to examine the PM_2.5_ concentration changes of different land-use types (3) to explore the impact of latent variables of urban factors on PM_2.5_ concentration. The results of the study will aid our understanding of the characteristics of the long-term trends of PM_2.5_ in China and have implications in designing effective strategies to control haze pollution.

## 2. Materials and Methods

### 2.1. Data Sources and Pre-Processing

#### 2.1.1. PM_2.5_ Data

The global PM_2.5_ concentration dataset was estimated by an optimal estimation algorithm that combines the Aerosol Optical Depth (AOD) obtained from multiple satellite products (MODIS, MISR, and SeaWiFS) with monitored surface PM_2.5_ concentration. The estimation algorithm was implemented through the GEOS-Chem model [11,12,13]. The resultant PM_2.5_ estimates were highly consistent (R^2^ = 0.81) with out-of-sample cross-validated PM_2.5_ concentrations from monitors [12].

The annual PM_2.5_ concentrations in China were retrieved from the global PM_2.5_ concentration with a grid spatial resolution of 0.01° [12,14,15,16]. The data were resampled to a grid scale of 1 km to match the data of land use. To address the uncertainty of abnormal values related to PM_2.5_ remote sensing inversion, previous studies often adopt an average taken across many years. PM_2.5_ remote sensing data therefore need to be preliminarily processed. The present study adopted the statistical method of using a 3-year average as the annual average.

#### 2.1.2. Land Use Data

Data of land use in China were provided by the Data Center for Resources and Environmental Sciences, Chinese Academy of Sciences (RESDC) [17]. The dataset having seven types of land use: agricultural land (Agr); forests (For); grassland (Gra); wetlands (Wet); built-up land (Bui); unused land (Unu); and ocean. With consideration of the proportions and potential for contribution to PM_2.5_ emissions, we selected the former six types.

#### 2.1.3. Administrative Boundary, Social and Economic Data

The administrative boundary layer with a scale of 1:250,000 was obtained from RESDC [17]. The national social and economic data (e.g., urban built-up area, gross domestic product (GDP), urban permanent population) in Chinese cities at prefecture level were collected from the China City Statistical Yearbook 2016, and some cities’ Statistical Yearbook and Statistical Bulletin 2016 data were also collected.

### 2.2. Methodology

#### 2.2.1. Trend Analysis of the PM_2.5_ Concentration

A least-squares model was used to demonstrate the trend of the PM_2.5_ concentration in China. Unary linear regression using the model reflected the trend of the PM_2.5_ concentration over time. The slope of the regression represents the change in concentration over time, with a positive value indicating an increasing trend. The slope is expressed as:

(1) $$\mathrm{slope}=\frac{n*\sum_{i=1}^{n\;}{\mathrm{PM}}_{2.5_{i}}-\left(\sum_{i=1}^{n}i\right)\left(\sum_{i=1}^{n}{\mathrm{PM}}_{2.5_{i}}\right)}{n*\sum_{i=1}^{n}i^{2}-{\left(\sum_{i=1}^{n}i\right)}^{2}}$$

where PM_2.5_ is the cell data of the PM_2.5_ concentration, n (taken as 16 in this study) is the time span, and i is the time unit ranging from 1 to 16.

A Pearson’s correlation test was conducted on the PM_2.5_ concentration time series data to reduce the uncertainty in trend changes:

(2) $$r=\frac{\sum_{i=1}^{n}\left({\mathrm{PM}}_{2.5_{i}}-\bar{{\mathrm{PM}}_{2.5}}\right)\left(t_{i}-\bar{t}\right)}{\sqrt{\sum_{i=1}^{n}{\left({\mathrm{PM}}_{2.5}{}_{i}-\bar{{\mathrm{PM}}_{2.5}}\right)}^{2}}-\sqrt{\sum_{i=1}^{n}{\left(t_{i}-\bar{t}\right)}^{2}}}$$

where t denotes the time series from 1 to 16. Correlation coefficients range from −1 to 1.

In the following analysis, we consider a PM_2.5_ trend significant for a *p*-value of ≤0.05 and a correlation coefficient of |r| ≥ 0.5. The calculations of this part was created by software ArcGIS 10.3 (Environmental Systems Research Institute, Inc., Redlands, CA, USA).

#### 2.2.2. Model Building

The selected variables are all available in the studied cities, and have been cited to have direct or indirect effects on PM_2.5_ (Table 1). We selected the corresponding socio-economic variables from three aspects of urban spatial factors, demographic factors and economic factors [18]. Spatial factors indicates urban spatial characteristic in the process of urban development [19,20]. Demographic factors comprises residents’ activities and population numeric information that differs between rural and urban [21,22]. Economic factors contains indicators that can reflect urban economic activities [23]. Moreover, PM_2_._5_ pollution condition were measured by observed variables: the urban average PM_2.5_ concentration (PM_2.5_ mean) and urban maximum PM_2.5_ concentration (PM_2.5_ max). Urban built-up boundary was utilized to separate urban from non-urban areas [6]. PM_2.5_ mean represented the overall pollution level of a city, and the latter expressed the serious pollution condition of a city, which were commonly used types for pollution variables in SEM [10,24]. The above indicators are only selected representatives for this paper.

**Figure 1:**
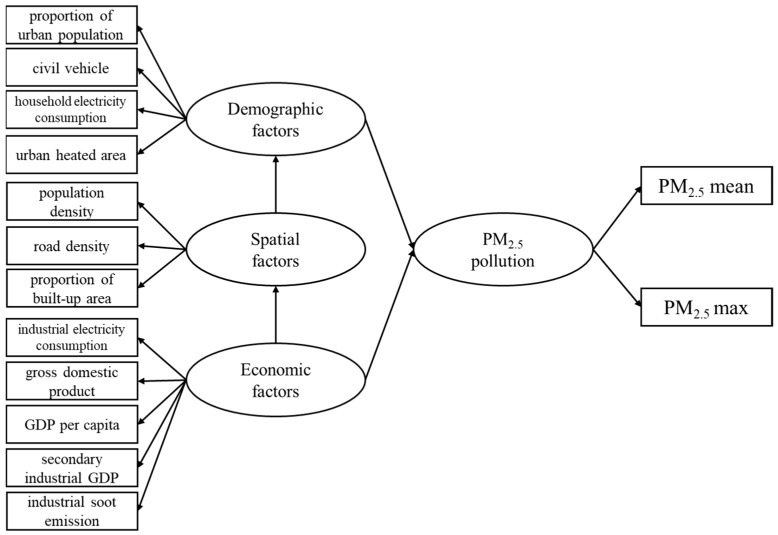
Initial frame for the SEM model. Variables in rectangles are observed variables; variable in ellipse are latent variables.

Previous studies have shown that the association between poor air quality and city size (weighed by urban population) was affected by the economic growth [25,26]. Namely, human activities and socio-economic activities within cities were the main direct acting factors on pollutant emissions. Therefore, we regarded the demographic and economic variables as the direct latent variables in the structure. Generally, the urban spatial characteristics have no direct effect on pollutant emission [9], so the spatial variable was treated as a mediating variable in the structure, and play an indirect effect on pollutant concentration. Above all, we constructed the structure of model (Figure 1).

All the twelve observed variables were added into the SEM (Figure 1), and according to the results, the two variables ISE and HA were removed because the regression weights did not pass the significance test during the model fitting. The SEM was fitted in software AMOS (version 21.0) (IBM, NY, USA).

## 3. Results

### 3.1. Spatial Pattern and Grade Variation in PM_2.5_ Concentration for 2000–2015

WHO standards set four levels of the PM_2.5_ concentration, namely the Air Quality Guide Line (AQG; <10 µg/m^3^), Interim Target 1 (IT-1; 35 µg/m^3^), Interim Target 2 (IT-2; 25 µg/m^3^), and Interim Target 3 (IT-3; 15 µg/m^3^). In estimating the spatial pattern of PM_2.5_ and its potential effect on the population, equal intervals of 15 µg/m^3^ were set to divide concentrations beyond 35 µg/m^3^.

**Figure 2:**
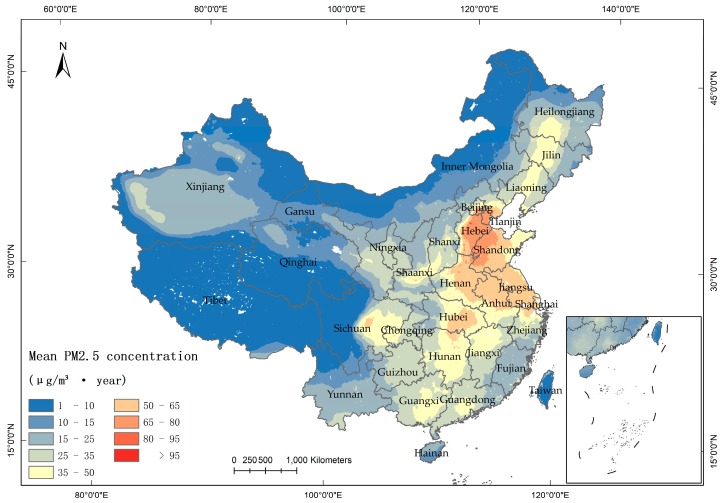
Spatial pattern of the mean PM_2.5_ concentration in China during 2000–2015.

Areas having a PM_2.5_ concentration beyond IT-1 were concentrated mostly in eastern China (Figure 2). Areas having a PM_2.5_ concentration beyond 80 µg/m^3^ had a punctuated distribution, and were mainly located in urban regions. Areas having concentrations between 65 and 80 µg/m^3^ were located in the Beijing–Tianjin–Hebei region (Jing-Jin-Ji) and in western Shandong Province. Areas having concentrations between 50 and 65 µg/m^3^ were concentrated in two regions, including the eastern part of China from Beijing and Hebei Province in the north to Shanghai, Jiangsu Province and Anhui Province in the south, and eastern Hubei Province. The second region was Chengdu in central Sichuan Province. Concentrations ranging from 35 to 50 µg/m^3^ covered 11 provinces, namely, Heilongjiang, Jilin, and Liaoning Provinces in northeastern China; Shaanxi Province in northwestern China; Hubei, Hunan, and Jiangxi Provinces in central China; Chongqing and Sichuan Provinces in southwestern China; and Guangdong and Guangxi Provinces in southern China. These were relatively seriously polluted areas. The regions with low PM_2.5_ concentrations at IT-1 and IT-2 levels were concentrated in the Taklimakan desert of Xinjiang, Yunnan, Fujian, and Hainan Provinces. The regions were lightly affected by PM_2.5_ at AQG and IT-3 levels were located in Taiwan, Tibet, Qinghai, and northern Inner Mongolia.

In addition to the above spatial pattern of pollutant concentration, we found some other interesting phenomena. Let us just assume that the regions covered by concentration higher than IT-1 (beyond 35 µg/m^3^) were heavily polluted areas. The distribution of the heavily and lightly polluted areas in Figure 2 were basically consistent with the Chinese population geographical distribution line —“Heihe-Tengchong line” proposed by Huanyong Hu in 1935. Therefore, it was reasonable to believe that the population differences and corresponding human activities played a primary role in the formation of PM_2.5_ spatial pattern. Besides, we also noticed that extreme pollution features appeared in the North China Plain and Sichuan Basin. Except the influence by human activities, topographic and geomorphologic features may also be a reason to the formation of spatial pattern.

**Figure 3:**
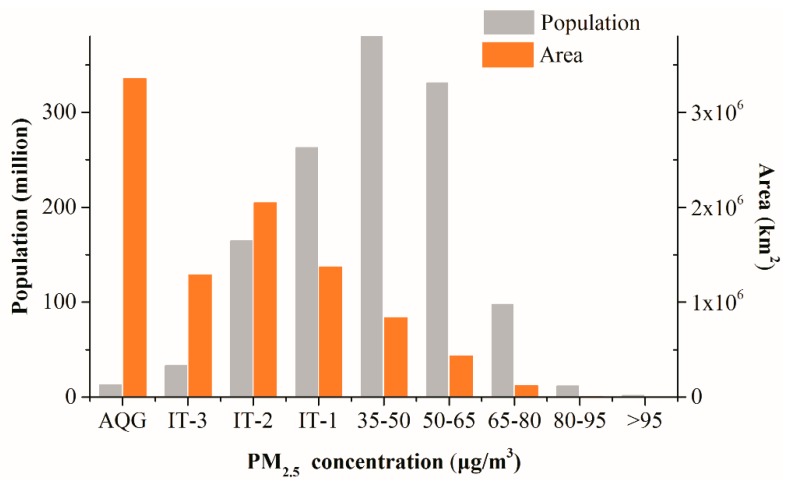
Area and population for each PM_2.5_ concentration level. Abbreviations for the PM_2.5_ standard levels used are as follows: AQG: Air Quality Guide Line; IT-1: Interim Target 1; IT-2: Interim Target 2; IT-3: Interim Target 3.

The exposure area and population were also examined. PM_2.5_ concentration beyond 80 µg/m^3^ covered the least population and area (Figure 3). In contrast, concentrations at the AQG level occupied the largest area of territory, but the area was mostly sparsely populated. From the changing of the area, we found that the interval of IT-3 was smaller than the intervals of other levels; therefore, if ignoring the abnormal fluctuation of the area bar at IT-3, the area tended to decrease with increasing PM_2.5_ concentration. In contrast, the population increased and reached a peak around the concentration of 50 µg/m^3^.

### 3.2. Trends of the PM_2.5_ Concentration for 2000–2015

**Figure 4:**
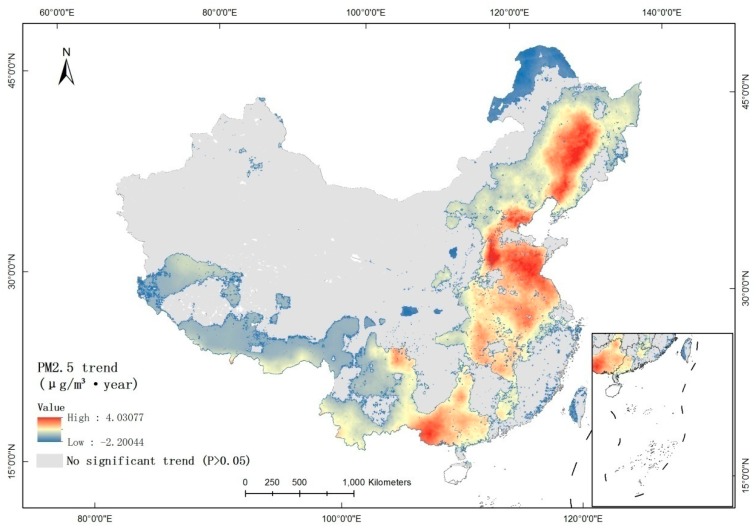
Significant trends of PM_2.5_ in China during 2000–2015.

Regions with a significant trend (*p* ≤ 0.05) were extracted (Figure 4). Regions having a significant positive trend were 1) northeastern China, including Liaoning, Jilin, and Heilongjiang Provinces, 2) the East China Plain, from Jing-Jin-Ji in the north to Anhui and Jiangsu Provinces, 3) southeastern Sichuan Province, and 4) southeastern Guangxi Province. Regions with a significant negative trend were concentrated in the conjunction of Shanxi, Sichuan, and Gansu Provinces, between Shanxi and Shaanxi Provinces, near the Great Xing’an Mountains, and in Taiwan Province.

**Figure 5:**
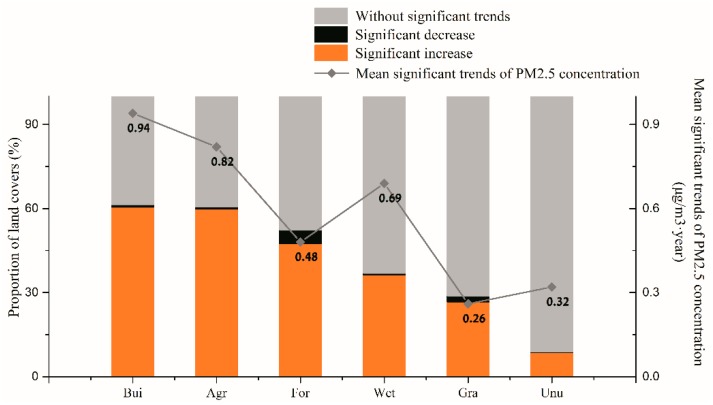
Mean trends of the PM_2.5_ concentration and proportion of different land use types. Abbreviations for the land use types used are as follows: Bui: built-up land; Agr: agricultural land (Agr); For: forests; Wet: wetlands; Gra: grassland; Unu: unused land.

The proportions of a significant positive trend for the six land use types were all apparently higher than the proportions of a significant negative trend (Figure 5). In the case of built-up land, 60.45% of the land had a significant increasing trend and only 0.95% had a significant decreasing trend. In the case of agricultural land, 59.74% of the land had a significant increasing trend and only 0.81% had a significant decreasing trend. Less than 50% of areas of forests, grassland, wetlands, and unused land had significant positive trends. Mean significant trends of the PM_2.5_ concentration were respectively 0.94 µg/m^3^·year and 0.82 µg/m^3^·year for built-up land and agricultural land, which were higher than those of the other land use types, while the lowest mean significant trend of the PM_2.5_ concentration was for grassland (0.26 µg/m^3^·year)

The built-up land is the main type of land use for the increase of PM_2.5_ concentration. Therefore, we believe that it is of great practical significance to study cities that are represented by the built-up land and to govern and control PM_2.5_ pollution.

### 3.3. Impact of Urban Socioeconomic Factors on the PM_2.5_ Concentration

**Figure 6:**
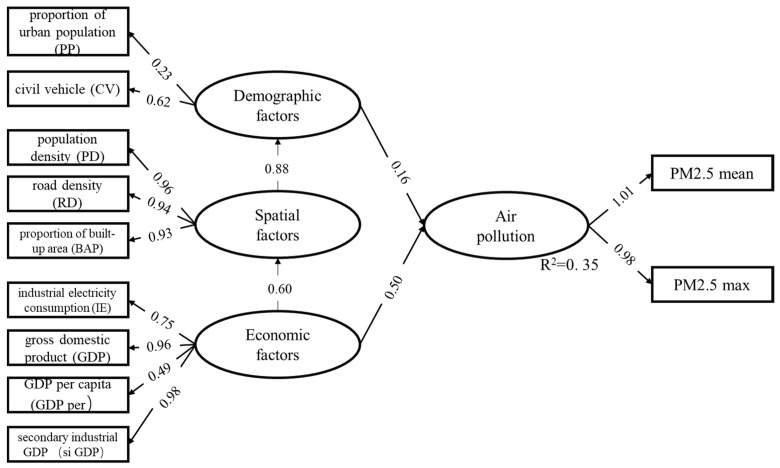
Results of the fitted model. The numbers in the figure on the paths represent the degree of contribution between variables.

To obtain a better-performing model, HE was removed because its inclusion led to poorer model fit (Table 2). Finally, nine observed variables were selected for SEM analysis (Figure 6).

Moreover, we used a multiple collinearity diagnosis to test the existence of collinearity between the observed variables. The results indicated that there was no strong collinearity (Tolerance >0.1 and VIF < 10) between the selected observed variables, hence the variables could be used for analysis.

Overall, the three latent variables included in our model could explain 35% of variations in PM_2.5_ pollution in 2015 (Figure 6). Economic factors influenced PM_2.5_ pollution more than spatial factors and demographic factors, and secondary industrial GDP were found to have the highest impact on PM_2.5_ pollution among all of the observed variables, while proportion of urban population and proportion of built-up area had the lowest (Table 3).

The direct and indirect influence of urban socioeconomic variables on PM_2.5_ pollution is shown in Table 3.

## 4. Discussion

### 4.1. Change of PM_2.5_ Concentration in the Past 15 Years

**Figure 7:**
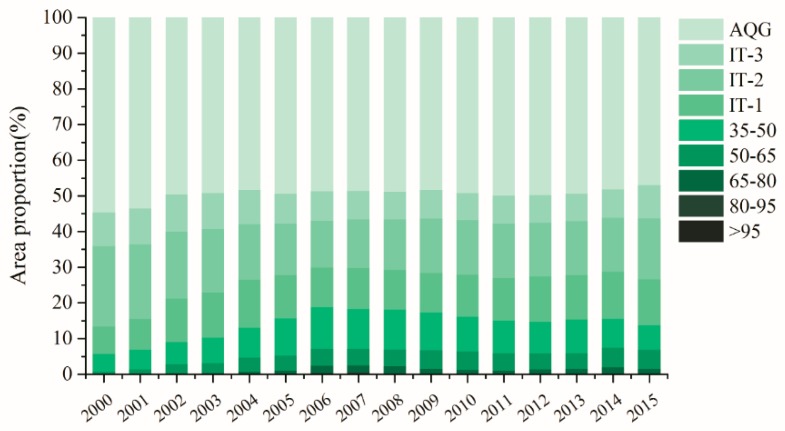
Area proportion of China under different PM_2.5_ concentration intervals from 2000 to 2015.

According to annual PM_2.5_ concentrations in China, the proportion of each interval was calculated (Figure 7). From the inter-annual variation, the area proportion in interval of AQG decreased from 54.67% to 46.93%, of which the changing trend from 2000 to 2004 was evident, and then, the changing tended to be stable. Accordingly, the area proportion of comparatively higher concentration (>35 μg/m^3^) increased from 5.75% in 2000 to 18.76% in 2006, then the changing trend slightly fluctuated and decreased to 13.78% in 2015, of which the intervals of 50–65 μg/m^3^ and 65–80 μg/m^3^ were respectively growing 0.25% and 0.09% a year on average. Though the area with interval of concentration >80 μg/m^3^ was heavily polluted, the area proportion was comparatively small, only around 0.4% from 2006 to 2008.

Since 2010, China has issued a series of policies on atmospheric environment governance, such as the *Environmental Air Quality Standard* and *Air Pollution Prevention and Control Action Plan* [36]. From Figure 7, we found that the area of comparatively higher concentration region had reduced, but fluctuated around 2013, and many people were still influenced in these regions from the preceding results. Therefore, although Chinese government and institutions have been aware of the importance of governing the atmospheric environment, it still has a long way to go to achieve effective governance outcomes.

### 4.2. Land Use and PM_2.5_ Pollution

**Figure 8:**
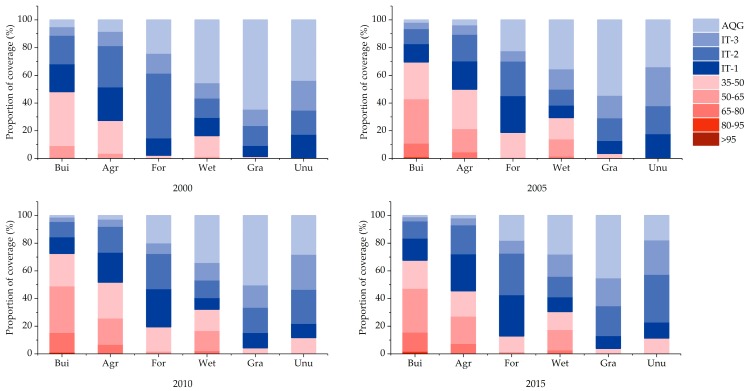
PM_2.5_ concentration of different land use types in the year of 2000, 2005, 2010 and 2015.

According to source apportionment models, the major PM_2 5_ sources in China have been identified to be coal combustion, motor vehicle emissions, industrial sources, soil dust [37,38], etc. It was shown that anthropogenic activities were the main contributors to the PM_2.5_ pollution. In Figure 8, we calculated the PM_2.5_ concentration of different land use types in the year of 2000, 2005, 2010 and 2015. We found that although the proportion of concentration levels fluctuated between different years, the basic pattern was consistent, that is, the proportions of high concentration (beyond 35 µg/m^3^) pollutants in built-up land were the highest. Meanwhile, according to the results in Figure 5 above, built-up land was also the type of land use with the most significant increasing trends in pollutant concentration in the past 15 years. Although the average changing trend and proportion of significant increasing area of PM_2.5_ concentration in agricultural land were also relatively large than the others, the proportion of PM_2.5_ with high concentrations in the land use type was still lower than that in built-up land. Therefore, it could be considered that cities centered on the built-up land were the most important source of PM_2.5_ pollution in China. At the same time, we should also pay attention to the pollution features of the types of ecological land such as forests and grassland. The rational layout of ecological land within city may play a positive role in improving the regional pollution condition.

### 4.3. Urban Factors and PM_2.5_ Pollution

In this study, economic factors contributed the most to the three urban latent variables, and related studies also found similar results. Jiang quantified the relative contribution of different socioeconomic factors to PM_2.5_ concentration using SEM and found that industrial activities contributed more to PM_2.5_ pollution than city size and residents’ activities. Though a city’s or region’s economic growth could be influenced by various factors, industrial contributions were still the priority drivers to most cities in China [39]. According to our results, industrial electricity consumption, GDP, GDP per capita, and secondary industrial GDP all contributed to the increase in PM_2.5_ concentration; GDP and secondary industrial GDP had the strongest impact strength. It was because that secondary industrial activities, such as industrial production and construction project, consumed a large amount of energy and resources, were often the main sources of particulate matters [40]. At present, China’s economic growth is still mainly driven by the secondary industry. To realize the transformation of industrial structure and reduce the over-dependence of regional economic growth on the secondary industry may will be a feasible measure to slow down the increasing of regional pollutant concentration. In addition, comparing the differences in results of GDP and GDP per capita impact on PM_2.5_ concentration, the overall intensity of economic activities in an area had a stronger impact on PM_2.5_ pollutants. Moreover, industrial electricity consumption in a region could comprehensively indicate the scale of economic activities in a city. As thermal power remains China’s main source of electricity, and the process would produce PM_2.5_ [41].

In the latent variables of demographic factors, the number of civil vehicles was the biggest contributor to PM_2.5_ concentration, and also played moderate influence among all observed variables. This result further confirmed the contribution of traffic emission to urban air pollution [42,43]. At the same time, with the growth of the urban economy, people’s purchasing power continued to increase, leading to the annual increase in the ownership of civil vehicles; the quality of car fuel and incomplete combustion and other problems, were still a serious issue for the impact of traffic emissions on urban air pollution in China. In some studies, winter heating was confirmed as a major driving force to the PM_2.5_ pollution in winter [31]; however, in this study, the indicator heated area was removed in the model fitting process, which was mainly because the use of annual average pollutant concentration data would weaken the contribution of winter heating to PM_2.5_ concentration; meanwhile, the Chinese heating area has the obvious regional differentiation, and the heating mechanism was also different between cities [44]. Therefore, only using heating area to measure the contribution of the winter heating of PM_2.5_ pollution may be biased. To accurately analyze the influence of winter heating on regional PM_2.5_ concentration, studies should be carried out on pollutants in specific regions and seasons.

Spatial factors affected the PM_2.5_ condition mainly through the demographic factors, which had weakest effect among latent variables. Larger urban built-up area, higher population density and denser road network density will lead to more human activities and more resource consumption, thus indirectly affecting PM_2.5_ concentration [45,46].

### 4.4. Limitations

In our study, we use remote sensed surface PM_2.5_ concentration to explore the spatiotemporal PM_2.5_ pattern and the contribution of urban socioeconomic factors. However, we still need to address some limitations in the study.

First, because the surface PM_2.5_ concentration was an annual average concentration, some of the region’s serious pollution events would be weakened. However, by comparing the relevant research results, we believe that our results are basically reliable. To further analyze the relationship between urban socioeconomic factors and air pollution, a more detailed time and space scale can be selected to study the relationship in the future.

Second, the indicators selected in this study were generally and widely cited, and which were easily acquired from statistic yearbooks. Some new emerging data sources and data that may affect or reflect the characteristics of city, but are less referenced, may play an important role in future studies.

Third, the socioeconomic variables selected in our model explained 35% of variation in urban PM_2.5_ pollution. That indicated that other factors besides the urban factors also have effects on urban PM_2.5_ pollution. Weather conditions, topographic and geomorphologic features and outside sources of pollutants could be important factors. Different meteorological factors such as wind speed, relative humidity, and temperature were all tightly correlated with air pollution in urban areas. Meanwhile, regional transport of pollutants was also an important source [47,48]. It also shows that the governance of PM_2.5_ pollution is a huge project of the whole. We should not only in terms of policies to control human contribution to pollution, but also in the long term to consider the adverse impact of human actions on all environmental factors.

### 4.5. Action and Policy Recommendations to Control PM_2.5_ pollution

There is an urgent need to develop a series of short-term actions and long-term policies to mitigate the current PM_2.5_ pollution condition in China. Policy-makers should fully consider the reducing abilities of ecological land, such as forests and grassland, on the concentration of particulate matters [49]. Moreover, controlling the expansion of built-up land and reasonably increasing the area of green space within cities are strongly suggested.

How to control urban factors to reduce PM_2.5_ pollution is obviously an effective but complex problem. Combined with our findings and previous research results, we discreetly put forward the following recommendations to control PM_2.5_ pollution: (1) vigorously develop urban public transport, reduce the use of private vehicles and improve the quality of gasoline; (2) regional economic growth should avoid excessive dependence on the secondary industry, and consider the feasibility of regional industrial transformation; (3) development and application of new energy sources to replace traditional fuels; (4) improve laws and regulations on punishment of illegal discharge, increase the cost of violation on enterprises; (5) strengthen public education to enhance environmental protection awareness; (6) identify the main causes of pollution in different regions and formulate targeted treatment actions [50,51].

## 5. Conclusions

This study analyzed spatial patterns and temporal changes of PM_2.5_ concentration in China from 2000 to 2015 based on a long sequence of PM_2.5_ concentration remote-sensing spatial data. The PM_2.5_ concentration changes within 15 years of different land use types were also examined. On that basis, we selected urban socioeconomic factors from spatial, demographic and economic aspects to explore the contributions of urban factors to PM_2.5_ in China. The study led to the following conclusions.

Seriously polluted locations (>35 µg/m^3^) in China were mainly concentrated on the eastern plain and in Sichuan Province. High concentrations were mainly distributed in densely populated areas. The trends of the PM_2.5_ concentration on the eastern plain, northeast China, Sichuan, and Guangxi Provinces were positive. Meanwhile, increasing trends being strongest for built-up land and agricultural land, and decreasing trends were strongest for forests and grassland, but the overall trend was still growing. The economic factors contributed most to PM_2.5_ pollution, followed by demographic factors and spatial factors. Among all variables, the secondary industrial GDP was found to have the highest impact on PM_2.5_ pollution.

Based on the above results, PM_2.5_ pollution was still an important pollution problem in China at present and even in the future. Decision-makers urgently need to formulate actions and policies from various aspects such as sources, influence factors, mitigation methods, etc. Meanwhile, residents should enhance environmental awareness and make contributions to control air pollution in China.

## Figures and Tables

**Table 1:** Summary of the results of previous correlation studies.

Latent Variables	Observed Variables	Results of Previous Studies
Spatial factors	proportion of built-up area (BAP)	Positive, R^2^ = 0.36, *p* < 0.05, Pearson correlation analysis [6] Positive, coefficient: 0.116, regression model [27]
	population density (PD)	Positive, coefficient: 0.14, R^2^ = 0.82, *p* < 0.05, regression model [28]
	road density (RD)	Positive, coefficient: 0.48, *p* < 0.05, regression model [29]
Demographic factors	household electricity consumption (HE)	Positive, 20% decrease in the PM_2.5_ with 2.2% decrease of electricity consumption, generalized linear model [30]
	urban heated area (HA)	Positive, R^2^: 0.51, *p* < 0.05, regression model [31]
	proportion of urban population (PP)	Positive, R^2^ = 0.99, *p* < 0.05, Pearson correlation analysis [6]
	number of civil vehicles (CV)	Positive, R^2^: 0.65, *p* < 0.05, regression model [2] Positive, coefficient: 0.10, Geographically weighted regression (GWR) model [32]
Economic factors	industrial electricity consumption (IE)	Positive, 379Mt PM_2.5_ emission, statistical description [33]
	industrial soot emission (ISE)	Positive, coefficient: 7.05664 × 10^−5^, *p* < 0.01, regression model [29]
	Gross Domestic Product (GDP)	Positive, R = 0.58, Geographically Weighted Regression [34]
	GDP per capita (GDP per)	Negative, coefficient: 0.39, R^2^ = 0.80, *p* < 0.05, regression model [28] Positive, coefficient: 0.32, *p* < 0.1, regression model [5]
	secondary industrial GDP fraction (si GDP)	Positive, coefficient: 0.34, R^2^ = 0.68, *p* < 0.05, regression model [28] Positive, coefficient: 0.36, Geographically weighted regression (GWR) model [32] Positive, coefficient: 0.52, regression model (R^2^ = 0.47) [35]

**Table 2:** Comparison of fitting results among models.

Models	Remove	χ^2^	GFI	A-GFI	AIC	BIC	CFI
Original		346.80	0.76	0.57	416.80	527.76	0.89
**Demographic A**	**HE**	**237.67**	**0.81**	**0.62**	**301.67**	**403.12**	**0.91**
Demographic B	PP	321.35	0.78	0.56	385.35	486.80	0.89
Demographic C	CV	315.03	0.75	0.55	375.03	470.14	0.89
Spatial A	PD	319.70	0.76	0.54	381.70	479.98	0.88
Spatial B	RD	320.17	0.75	0.51	384.17	485.62	0.88
Spatial C	BAP	287.85	0.78	0.56	353.85	458.47	0.89
Economic A	IE	293.04	0.80	0.61	357.04	458.49	0.90
Economic B	GDP_per	294.87	0.80	0.62	356.87	455.16	0.90
Economic C	GDP	257.74	0.82	0.61	327.74	438.70	0.90
Economic D	si GDP	261.77	0.81	0.64	325.77	427.22	0.90

Note: Abbreviations: chi-square (χ^2^); goodness-of-fit index (GFI); adjusted goodness-of-fit index (A-GFI); Akaike information criterion (AIC); Bayesian information criterions (BIC); comparative fit index value (CFI). Household electricity consumption (HE); proportion of urban population (PP); number of civil vehicles (CV); population density (PD); road density (RD); proportion of built-up area (BAP); industrial electricity consumption (IE); Gross Domestic Product (GDP); GDP per capita (GDP per); secondary industrial GDP fraction (si GDP). Bold words represent the parameters of final selected model.

**Table 3:** The influence of urban socioeconomic variables on PM_2.5_ pollution in China.

Latent Variables	Observed Variables	Normalized Coefficient		
		Direct	Indirect	Total
**Demographic**		**0.179**	**0.000**	**0.179**
	PP	0.048	0.000	0.048 ^w^
	CV	0.131	0.000	0.131 ^m^
**Spatial**		**0.000**	**0.158**	**0.158**
	PD	0.000	0.053	0.053 ^w^
	RD	0.000	0.053	0.053 ^w^
	BAP	0.000	0.052	0.052 ^w^
**Economic**		**0.568**	**0.095**	**0.663**
	IE	0.134	0.022	0.156 ^m^
	GDP per	0.087	0.015	0.102 ^m^
	GDP	0.172	0.029	0.201 ^s^
	si GDP	0.175	0.029	0.204 ^s^

Note: The superscript of ‘w’ meant weak influence; ‘m’ meant moderate influence, ‘s’ meant strong influence. These were the relative strength in the study. Bold numbers represent the influence coefficients of the three latent variables.
